# The land use changes and its relationship with topographic factors in the Jing river catchment on the Loess Plateau of China

**DOI:** 10.1186/2193-1801-2-S1-S3

**Published:** 2013-12-11

**Authors:** Zhi Li, WenZhao Liu, FenLi Zheng

**Affiliations:** College of Natural Resources and Environment, Northwest A & F University, Yangling, Shaanxi 712100 China; Institute of Soil and Water Conservation, CAS & MWR, Yangling, Shaanxi China

**Keywords:** Land use changes, topographic factors, the Loess Plateau, transfer direction

## Abstract

A series of soil conservation measures have been carried out to reduce soil loss on the Loess Plateau of China since 1950s, and the biologic measures were implemented according to topographic factors such as slope and elevation; therefore, the changes in topographic factors of land use can indicate the effects of the biologic measures. The objectives of this study were to (i) analyze the land use changes in the Jing River catchment during 1986-2000 and to (ii) examine the effects of biologic measures through relating land use changes with topographic factors. During 1986-2000, the dominant land use types were farmland and grassland (88% of the whole catchment). Compared with 1986, farmland and forest decreased while grassland and construction land increased with little changes in water and unused land. Three main conversion types occurred, i.e. the mutual conversion between forest and grassland, the mutual conversion between farmland and grassland, and farmland converted to other types. The elevation of farmland, forest, construction land and water increased, while that of grassland and unused land decreased. The mean slope gradient of each land use type changed little except for unused land. The above results suggested farmland has greatly decreased on tableland region due to the increase in construction land, forest has moved to gully region while grassland has increased despite elevation and slope. The land use in the Jing River catchment during 1986-2000 was changing to a more reasonable spatial pattern.

## Introduction

The Loess Plateau (6.4 × 10^5^ km^2^) is situated in north China. It is covered with highly erodible aeolian deposits. Most areas belong to semiarid to sub-humid climate with most precipitation falling in summer months largely in forms of heavy storms. Canopy cover degree is generally low, and land use is predominantly cultivated cropland and improved grassland. The Yellow River, which has highest sediment concentration in the world [1], runs through the Loess Plateau. Due to the above situation, the Loess Plateau has become one of the most severely eroded areas in the world [2, 3].

Since 1950s, a series of conservation measures including replanting trees and improving grassland, constructing of terraces and sediment-trapping dams were implemented. Among these measures, returning steep farmland to forest and grassland was carried out in most areas to improve the vegetation over. Steep farmland accounts for about 50% of total farmland area on the Loess Plateau, and this situation is worse in the hilly-gully area, where steep farmland covers 70%-90% of the total farmland area and farmland with slopes over 25° accounts for 15%-20% of the total farmland area [4]. Thus, vegetation construction would not only change the spatial distribution of land use, but also alter the slope of each land use type [5, 6]. For example, 25° was usually taken as the critical slope gradient of returning farmland to forest and grassland, which might decrease the slope of farmland and increase that of forest and grassland.

With the implementation of conservation measures, human choices of land use have also changed greatly due to the impacts of elevation [5, 6]. For example, farmland abandonment often occurred on the highest or lowest and the steepest areas while construction land often moved to the flattest areas with good traffic condition and water supplying. Besides, as climatic variations due to elevation differences between ridges and valley bottoms influence the land use types [7], vegetation construction would consider more about the suitability of plants, for example, forest in the Loess Plateau often moved to the valley with relative rich water now.

Therefore, analyzing the relationships between land use and topographic factors can reflect the effect of vegetation constructions and guide future vegetation restoration. However, this kind of this study is less common. The objectives of this study were to (i) detect the land use changes during 1986-2000 in the Jing River catchment on the Loess Plateau and to (ii) analyze the relationship between land use and topographic factors.

## Materials and methods

### Study area

**Figure 1:**
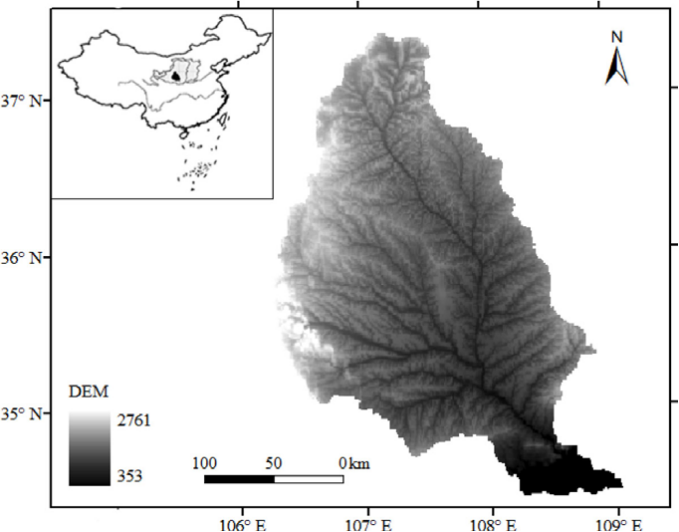
**The location of the Jing River catchment**.

The Jing River is a second order tributary of the Yellow river; the catchment is located in southern Loess Plateau of China (Figure 1). The catchment covers an area of 45,421 km^2^. The average annual precipitation in the catchment is 542.1 mm and the average annual temperature is 9.0 ^°^C. The southern part is more warm and wet than the northern part. The landscape is mainly forest-steppe ecotone. The soil is predominantly silt loam with silt content greater than 50%. The population exceeds 6.2 millions. The elevation varies during 353-2761 m. The erosion rate of the Jing River catchment is 5,015 t km^-2^ a^-1^, and the area subject to water erosion is 33,220 km^2^, which accounts for 73% of the whole catchment [8].

### Data description

Two land use maps of 1986 and 2000 (1:100,000), provided by the Environmental and Ecological Science Data Center for West China, were used to analyze the land use changes. The land use was classified into six categories: farmland, forest, grassland, water, construction land (areas for residence, traffic, industry and mining), and unused land.

Elevation and slope gradient was chosen as the indices of topography to study their relationship with land use. GDEM (Global Digital Elevation Model) of 30 m, a product of Trade and Industry of Japan (METI) and the National Aeronautics and Space Administration (NASA), was used to provide the elevation. The elevation was classified into three categories: <1000 m (mainly gully region), 1000-1500 m (mainly tableland region), and >2000 m (mainly hilly region). A slope map was generated through DEM surface analysis operation of ArcGIS 9.2 and the slope gradient was classified into three categories: gentle (<15°), intermediate (15-25°), and steep (>25°) in accordance with the national policy on converting steep lands to other uses.

### Spatial analysis

Spatial analysis was carried out by an overlaying operation of ArcGIS 9.2 to study the land use changes over time and to assess the relationship between land use and elevation or slope gradient. Through intersecting the two land use maps (1986 and 2000), a land use transformation map was extracted and used for subsequent transformation matrix analysis. Then several indices including change magnitude, speed and types were used to quantify land use changes. Change magnitude is the difference of area between the beginning and the end of the study period for each land use type. Change speed is usually quantified by the following equation:

where *K*_*i*_ is the change speed for the *i*th type land use; *U*_*b*_ and *U*_*a*_ is the area of a certain land use type at the beginning and end of the study period; *T* is the time duration of the research. The change speed is thus defined as the change area per year, positive value refers to upward trend and negative value refers to downward trend.

To analyze the relationship between land use and topographic factors, the two land use maps were combined with DEM or slope gradient map through the grid extension of Arc/Info 9.2. All maps were grid format and the cell size was 30 m×30 m. Through exporting the attribute table of the combined maps and calculating the elevation and slope gradient of different land use type in different year, the effects of biologic measures can be detected.

## Results and discussion

### Changes in land use

During 1986-2000, the dominant land use types in the Jing River catchment were farmland and grassland (Table 1), which accounted for about 88% of the entire area. Two main trends existed in land use changes, i.e. the decrease in farmland and forest, and the increase in grassland and construction land. Farmland and forest decreased by 85.1 and 123.8 km^2^, respectively; while grassland and construction land increased by 118.8 and 95.2 km^2^, respectively; water body and unused land decreased slightly. The changes in each land use type were the combined effects of two processes: conversion to other types and conversion from other types. For example, the area of grassland converted from other land use types (202.9 km^2^) was more than that of forest converted to other types (184.6 km^2^); however, the total change area of grassland (118.8 km^2^) was less than that of forest (123.8 km^2^) due to the balancing effect of the other process. In terms of change magnitude, land use was changing in the order of forest > grassland > construction land > farmland > water > unused land. However, the change speed was in the order of unused land > construction land > forest > water > grassland > farmland.

**Table 1:** Land use structure and their changes during 1986-2000 in the Jing River catchment

Land use	1986		2000		Unchanged area	Changed area	Converted to other types	Converted from other types	Change speed
	(km^2^)	(%)	(km^2^)	(%)	(km^2^)	(km^2^)	(km^2^)	(km^2^)	(%)
Farmland	20360.6	44.83	20275.5	44.64	20209.6	-85.1	151.0	66.0	-0.03
Forest	4313.6	9.50	4189.8	9.22	4129.0	-123.8	184.6	60.8	-0.19
Grassland	19843.8	43.69	19962.6	43.95	19759.7	118.8	84.1	202.9	0.04
Water	205.1	0.45	201.3	0.44	199.7	-3.8	5.4	1.6	-0.12
Construction land	695.4	1.53	790.6	1.74	695.4	95.2	0.0	95.2	0.91
Unused land	2.4	0.01	1.0	0.002	1.0	-1.4	1.4	0.0	-3.84

Table 2 lists the main types of land use changes. Overall, the land use in the Jing River catchment changed little from 1986 to 2000, and only about 0.9% of the entire catchment has underwent type conversions. There were mainly six conversion types whose areas were more than 10 km^2^. The changes fell into three groups: (i) the mutual conversion between forest and grassland, (ii) the mutual conversion between farmland and grassland, and (iii) farmland converted to other land use types.

**Table 2:** Change types of land use during 1986-2000 in the Jing River catchment

	Area (km^2^)	Changes relative to the entire area (%)	Changes relative to each land use type (%)
Forest to grassland	181.9	0.40	4.22
Farmland to construction land	92.6	0.20	0.45
Grassland to farmland	59.2	0.13	0.30
Farmland to forest	37.5	0.08	0.18
Grassland to forest	22.5	0.05	0.11
Farmland to grassland	20.1	0.04	0.10

### Land use changes in relation to elevation

The elevation distribution of each land use type is shown in Table 3. The elevation in the Jing River catchment was mostly over 1000 m. The area with elevation during 1000-1500 m and over 1500 m accounted for 62% and 31% of the whole catchment, respectively. The mean elevation of each land use type was in the order of water and grassland (about 1100 m) < construction land (about 1200 m) < farmland (about 1310 m) < forest and unused land (over 1500 m). The elevation distribution of each land use type was different. About 60% of farmland, forest, grassland and construction land distributed within 1000-1500 m, about 30% of farmland, forest and grassland distributed over 1500 m; however, only 10% of water and construction land located over 1500 m and over 20% of both types distributed below 1000 m. The elevation distribution was related to the characteristics of each land use type. For example, as river run across hilly region with low elevation, construction land also located lowly due to the convenience of getting water for people.

**Table 3:** Land use changes in relation to elevation during 1986-2000 in the Jing River catchment.

		Farmland	Forest	Grassland	Water	Construction land	Unused land
Mean elevation (m)	1986	1317	1520	1065	1104	1200	1544
	2000	1314	1495	1098	1082	1175	1617
	Changes	3	25	-33	23	26	-72
1986 (km^2^)	<1000	1901	119	662	86	157	0
	1000-1500	12833	2645	12284	103	475	0
	>1500	5653	1518	6903	17	63	2
2000 (km^2^)	<1000	1870	122	663	85	183	0
	1000-1500	12751	2569	12383	101	537	0
	>1500	5680	1466	6922	16	71	1
Land use changes (km^2^)	<1000	-31	3	2	0	26	0
	1000-1500	-82	-76	99	-2	61	0
	>1500	27	-52	19	-1	8	-1

In general, two trends existed in elevation changes of land use, i.e. the increase in elevation of farmland, forest, water and construction land, and the decrease in that of grassland and unused land. The changes in elevation for each land use type were significant. Specifically, land use changes mainly occurred during 1000-1500 m. Farmland has substantially decreased by 113 km^2^ below 1500 m while increased by 27 km^2^ over 1500 m, indicating that a large number of farmland in gully region was transferring to other land use types. Forest increased by 3 km^2^ below 1000 m and decreased by 128 km^2^ over 1000 m, suggesting that forest were transferring to gully regions. Grassland and construction land increased despite the elevation. Grassland tended to increase in regions with high elevation while construction land increased preferably in tableland and rive valley with low elevation. The decrease in water mainly occurred over 1000 m. The above changes in elevation of land use were possibly caused by the substantial conversion from forest to grassland, and conversion from farmland to construction land (Table 2).

### Land use changes in relation to slope gradient

The mean slope gradient of each land use type was relatively small (Table 4) due to the predominant area of the tableland and river valley that were mostly flat. In general, the slope gradient of about 90% land was less than 15°, 7% land was during 15°-25°. The mean slope gradients of construction land, water and farmland were less than 10.7°, while those of forest, grassland and unused land were more than 13.5°. About 27% of farmland, 52% of forest, 43% of grassland, 12% percent of water and 9% of construction land distributed on areas with slope gradients over 15°. As 15° was recommended by some experts as the critical degree to return farmland to other land use types, there are great potential of reducing farmland to promote ecologic environment.

**Table 4:** Land use changes in relation to slope during 1986-2000 in the Jing River catchment

		Farmland	Forest	Grassland	Water	Construction land	Unused land
Mean slope gradient (°)	1986	10.71	16.68	14.78	6.35	5.62	13.54
	2000	10.74	16.69	14.78	6.40	5.58	15.90
	Changes	0.03	0.01	0.01	0.05	-0.05	2.36
1986 (km^2^)	<15°	14974	2052	11233	180	633	1.5
	15°-25°	4316	1579	6602	18	50	0.7
	>25°	1097	652	2013	7	13	0.1
2000 (km^2^)	<15°	14893	1986	11295	176	722	0.5
	15°-25°	4313	1529	6650	18	55	0.4
	>25°	1095	643	2023	7	14	0.1
Land use changes (km^2^)	<15°	-81	-66	62	-4	89	-1.1
	15°-25°	-2	-50	48	0	5	-0.3
	>25°	-2	-9	10	0	2	0

During 1986-2000, the mean slope gradient of each land use type changed little except for the increase in unused land. The changes in land use mainly occurred at area with slope gradient less than 15°. Farmland decreased by 81 km^2^ with slope less than 15° possibly because it was used as construction land (Table 2). Forest decreased while grassland increased despite slope gradient, which was possibly caused by the conversion from forest to grassland (Table 2). Unused land decreased in regions with small slope.

### Implication of land use changes and its relation to topographic factors

The transferring direction of land use in the Jing River catchment during 1986-2000 can be detected according to the above results. Farmland has decreased greatly on tableland, which was possibly caused by converting farmland to construction land and increasing farmland by constructing terraces. Forest have moved to gully region with low elevation and small slope gradient, indicating that the vegetation construction of the Loess Plateau was getting more rational and considering more about the suitability of planting trees. Grassland has increased at all elevation and slope, implying that the effects of returning steep farmland to grassland and converting from forest to grassland were significant. Construction land has increased despite elevation and slope due to population increase. Unused land has moved to hilly region with high elevation and big slope gradient, suggesting the development and utilization of land use are more intensive.

Overall, the land use in the Jing River catchment during 1986-2000 was changing to a more reasonable spatial pattern, which considered more about the suitability of plants and human needs. This kind of changes will surely benefit the vegetation construction and sustainable development of this region.

## Conclusion

The dominant land use types of the Jing River catchment were farmland and grassland. Overall, the land use changed little during 1986-2000, indicating that the soil conservations measures now implemented mainly focused on engineering measures, therefore, more attention should be paid to vegetation construction in the future. However, the land use distributions on elevation and slope gradients were becoming more reasonable, and the spatial pattern of land use were considering more about the suitability of plants and human needs. This kind of changes would indubitably promote the ecological environment and sustainable development of the region.
